# Seasonal dynamics of *Phlebotomus neglectus* (Diptera: Psychodidae) in cave microhabitats in Romania and the rediscovery of *Sergentomyia minuta* (Rondani, 1843) after 50 years

**DOI:** 10.1186/s13071-021-04985-y

**Published:** 2021-09-15

**Authors:** Cristina Daniela Cazan, Cintia Horváth, Luciana Cătălina Panait, Daniela Porea, Mihai Marinov, Vasile Alexe, Andrei Daniel Mihalca

**Affiliations:** 1 Molecular Biology and Veterinary Parasitology Unit, Faculty of Veterinary Medicine, University of Agricultural Sciences and Veterinary Medicine of Cluj-Napoca, CDS-9, Cluj-Napoca, Romania; 2 Department of Parasitology and Parasitic Diseases, Faculty of Veterinary Medicine, University of Agricultural Sciences and Veterinary Medicine of Cluj-Napoca, Cluj-Napoca, Romania; 3 Danube Delta National Institute for Research and Development, Tulcea, Romania

**Keywords:** Sand flies, *Phlebotomus balcanicus*, *Phlebotomus neglectus*, Periodicity, Abundance, Romania

## Abstract

**Background:**

In a countrywide study aiming to update the knowledge on diversity of sand fly species in Romania, a sand fly population was observed in an isolated system of cave microhabitats. The caves are located in the protected area of Canaraua Fetii, Dobrogea region, southeastern Romania. The highest sand fly diversity was recorded in this area between 1968 and 1970. This work presents a study conducted to estimate the seasonal variation of the sand fly species in correlation with the particular environmental factors of the isolated system of cave microhabitats.

**Methods:**

Sand flies were collected between May and October 2020 from one trapping site of interest in Canaraua Fetii. The trapping site consisted of a cave entrance. CDC miniature light traps and sticky traps were used to collect insects from the exterior walls of the cave entrance. Species identification of collected sand flies was done using morphological keys. Statistical analysis of the trapping and climatic data was performed.

**Results:**

From all collected sand flies, 99.7% (818/822) were *Phlebotomus neglectus*, 0.1% (1/822) *Ph. balcanicus* and 0.2% (2/822) *Sergentomyia minuta*. Sand fly activity was first observed on 2 July and last on 24 September. A monomodal abundance trend was present, with the peak activity between 16 and 17 July. The analysis of the climatic data showed correlations between the total number of captured sand flies and both average temperature and average relative humidity. The total number of collected specimens was statistically higher when CDC miniature light traps were used compared to sticky traps. The number of females on the sticky traps was significantly higher than the number of males on the same trap type. Compared with the sticky traps, significantly more males were collected by CDC miniature light traps. This is the first record of *Se. minuta* in Romania after 50 years of no records (despite the trapping effort of the last 5 years in the country). Also, *Ph. sergenti*, previously present in this location, was not found.

**Conclusions:**

In the investigated natural habitat, the diversity of the sand fly species appears to have changed, with the predominance of *Ph. neglectus* instead of *Ph. balcanicus* and *Se. minuta* (recorded as the two predominant species in 1968–1970). A monomodal abundance trend was observed as in other regions of the country. The sand fly activity in this particular cave microhabitat appears to be longer than in other regions in Romania. Longer sand fly activity increases the zoonotic risk of various pathogenic species’ transmission, with an impact on public health, as sand flies are important insect vectors.

**Graphical abstract:**

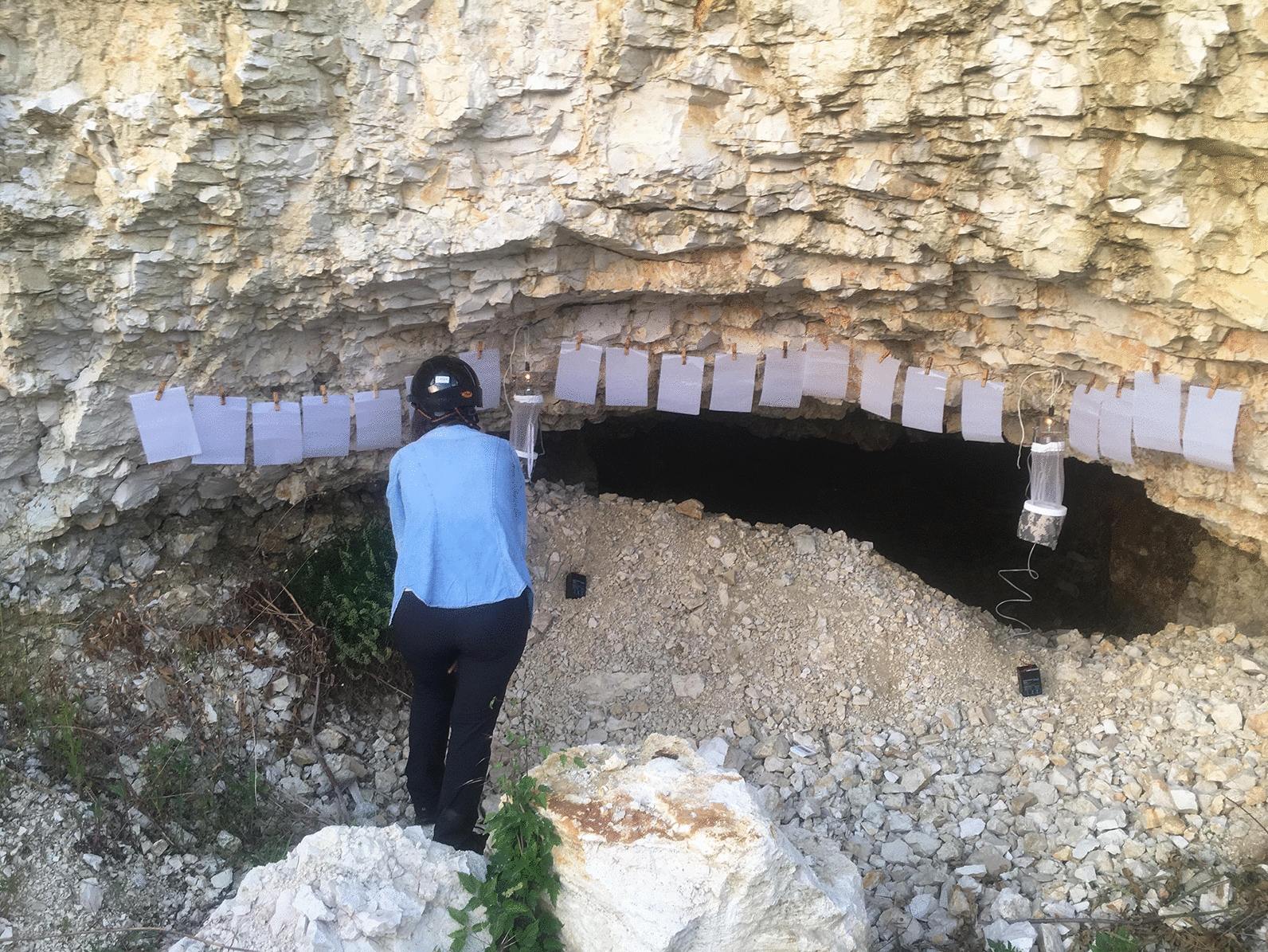

**Supplementary Information:**

The online version contains supplementary material available at 10.1186/s13071-021-04985-y.

## Background

In both the Old and the New World, sand flies (Diptera, Psychodidae, Phlebotominae) are important hematophagous insects of public health and veterinary concern [1]. The major vectorial role in the transmission of parasites of genus *Leishmania* (Kinetoplastida, Trypanosomatidae) gives sand flies an important status among other vector insects. Their vectorial role was also confirmed for other bacterial and viral pathogens [2].

In southern Europe, and more particularly in the Mediterranean basin, which is highly endemic for zoonotic visceral leishmaniasis in humans (VL) and canine leishmaniasis in dogs (CanL), both caused by *Leishmania infantum*, sand fly species are abundant [2]. In Romania, three sand fly species are confirmed vectors for *L. infantum*: *Phlebotomus perfiliewi*, *Ph. neglectus* and *Ph. balcanicus* [2–4]. The important vectorial role of sand flies and the permanent risk of new foci of disease emergence require permanent surveillance of vector presence, abundance and disease epidemiology, mainly at the limit of their distribution [5].

In Romania, eight sand fly species were recorded between 1910 and 1970, namely *Phlebotomus perfiliewi*, *Ph. neglectus*, *Ph. balcanicus*, *Ph. papatasi*, *Ph. alexandri*, *Ph. sergenti*, *Ph. longiductus* and *Sergentomyia minuta* [6]. The highest sand fly diversity recorded in the country between 1968 and 1970 was found in the protected natural habitat of Canaraua Fetii, Dobrogea region, southeastern Romania, with four sand fly species: *Ph. neglectus*, *Ph. balcanicus*, *Ph. sergenti* and *Se. minuta* [7]. In more recent studies conducted between 2013 and 2018, only five sand fly species were identified in Romania: *Ph. perfiliewi*, *Ph. neglectus*, *Ph. balcanicus*, *Ph. papatasi* and *Ph. sergenti* [3, 4]. Currently, Mehedinţi Plateau (southwestern Romania) appears to have the highest sand fly species diversity in Romania, with five species recorded [3]. To date, one single study has reported the seasonality of sand flies in Romania [4], which revealed a monomodal type of abundance trend for *Ph. perfiliewi*, with a single activity peak at the beginning of August, in the northeastern region of the country.

The present study was designed to re-evaluate the sand fly diversity and abundance in the protected natural area of Canaraua Fetii, a sylvatic area with a history of high sand fly diversity, and to describe the seasonal trends of sand flies in a cave microhabitat.

## Methods

### Study area and design

The study was conducted in the protected area of Canaraua Fetii in southeastern Romania (44.07302N, 27.64289E), where a population of sand flies has been observed during previous field studies [7]. The protected area is situated in the southwestern part of Dobrogea Plateau. It is a limestone canyon, carved by a former river among hills forming a plateau. It has deciduous forests on both sides and typical short-grass steppes on top. The valley is moist (a temporary brook crosses it, with slow-flowing water following rains), while the plateau is drier. Elevation is 100–130 m on the plateau and 18–26 m in the valley. A high diversity of animal species with important bird and bat populations is present [8].

**Fig. 1:**
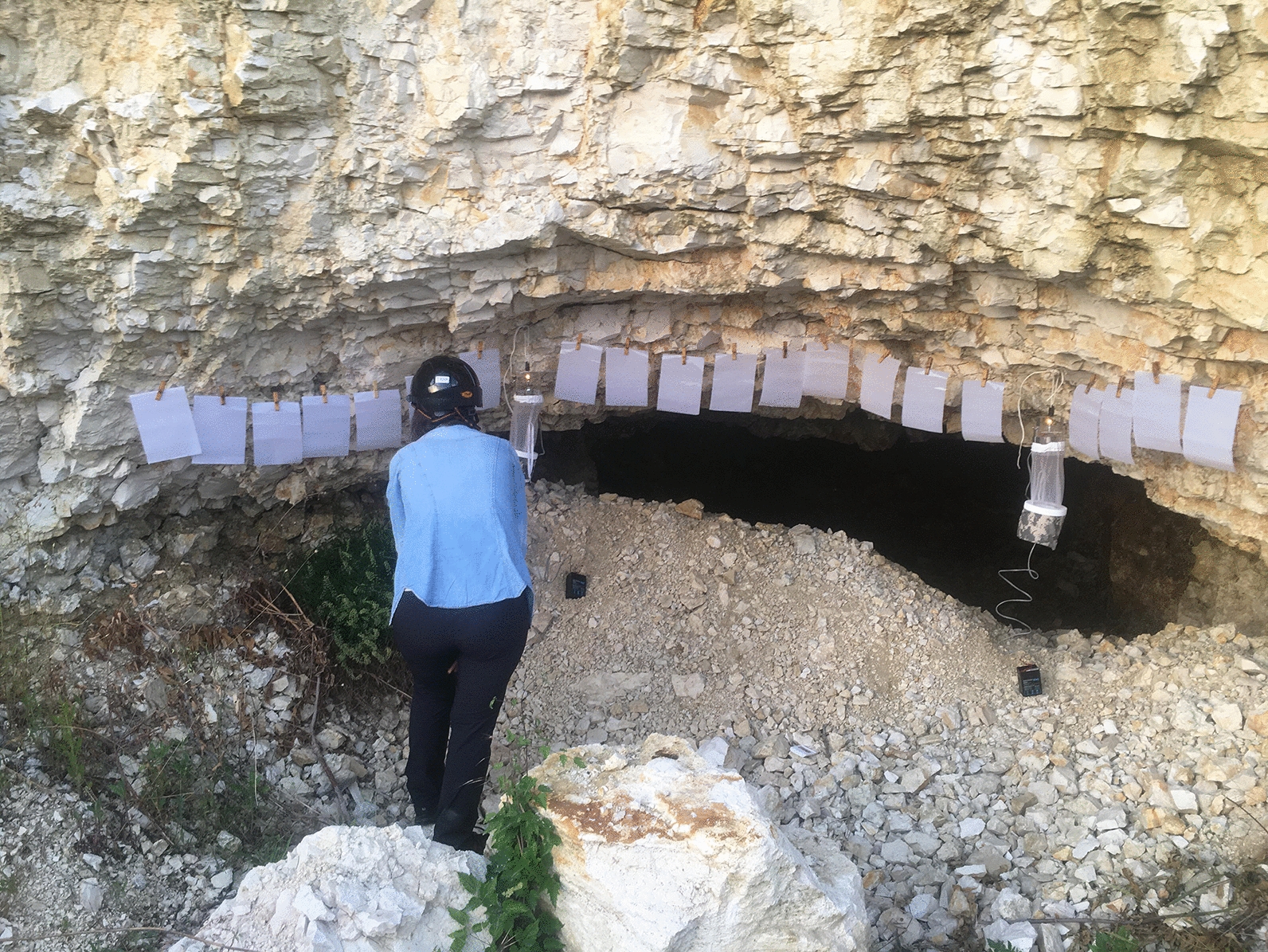
Trapping site in Canaraua Fetii, Dobrogea region, Romania

Between 21 May and 8 October 2020, insect collections were made using two CDC miniature light traps set 12 times for 2 consecutive nights on the exterior walls of the cave entrance. The light-attraction collections were complemented by using sticky traps as these were demonstrated to influence the proportions of males and females collected [9, 10]. Sticky traps consisted of A5 format white paper (148 × 210 mm) coated with castor oil; a fixed number of sticky traps per site (*n*  =  20) was set in each trapping site during the sampling period. All traps were set overnight (19:00–05:30 h) on the very same place for the entire study, close to the walls, at 1.5 m height from the ground (Fig. 1).

The frequency of insect collection was initially at 2-week intervals until the first positive collection. After that, the insect collection was performed weekly. After the last sampling in August, the collections were carried out again for 2 weeks in September and finally stopped after two consecutive negative samplings. Overall, there were 12 trapping periods (Additional file 1: Table S1).

The total number of light traps placed in the study site was 48 (2 CDC miniature light traps  ×  1 site  ×  2 consecutive nights  ×  12 sampling times). A total surface of 14.4 m^2^ [0.03 m^2^ (standard for A5)  ×  20 sticky traps  ×  2 (both sides of the paper)  ×  12 sampling times] of sticky traps immersed in castor oil was used.

### Species identification

After each trapping night, insects were collected, stored in 70% ethanol and transferred to the laboratory for species identification. Sand flies were separated from other insects. The heads and genitalia of each specimen were dissected and individually slide-mounted. The slide mounting was done in Swan solution (chloral hydrate/acetic acid/Arabic gum). Entomological keys [11–14] were used for species identification. The morphological identification of the species was based on specific features of the pharynx, cibarium and external genitalia in males and pharynx, cibarium and internal genitalia in females.

### Environmental data collection

The minimum, maximum, average daily temperatures and average relative humidity for the study locality were collected from the Romanian National Meteorological Administration (RNMA) for each day of the sampling period (see Additional file 2: Table S2).

### Data analysis

Shapiro-Wilk normality test was used to assess the distribution of data. The impact of environmental factors (average, minimum and maximum temperature, and relative humidity) on sand fly population dynamics was evaluated using Spearman’s correlation. The strength of correlation was assessed based on the Spearman correlation coefficient (*rs*) (0.00–0.19: very weak; 0.20–0.39: weak; 0.40–0.59: moderate; 0.60–0.79: strong; 0.80–1.0: very strong). Chi-square goodness of fit, Pearson’s chi-squared and nonparametric Mann-Whitney *U* tests were used to compare the number of collected sand flies by trap type, gender and sampling periods. Linear regression analyses were performed to establish the existence of a linear relationship between the number of collected sand flies and environmental factors. Prediction models for the presence or absence of phlebotomine sand flies, based on humidity and average temperatures, were performed using logistic regression. *P* values < 0.05 were considered statistically significant. Data were analyzed using R software v. 4.0.5.

### Mapping

The map was generated using QGis 3.6.2 software (http://www.qgis.org).

## Results

**Fig. 2:**
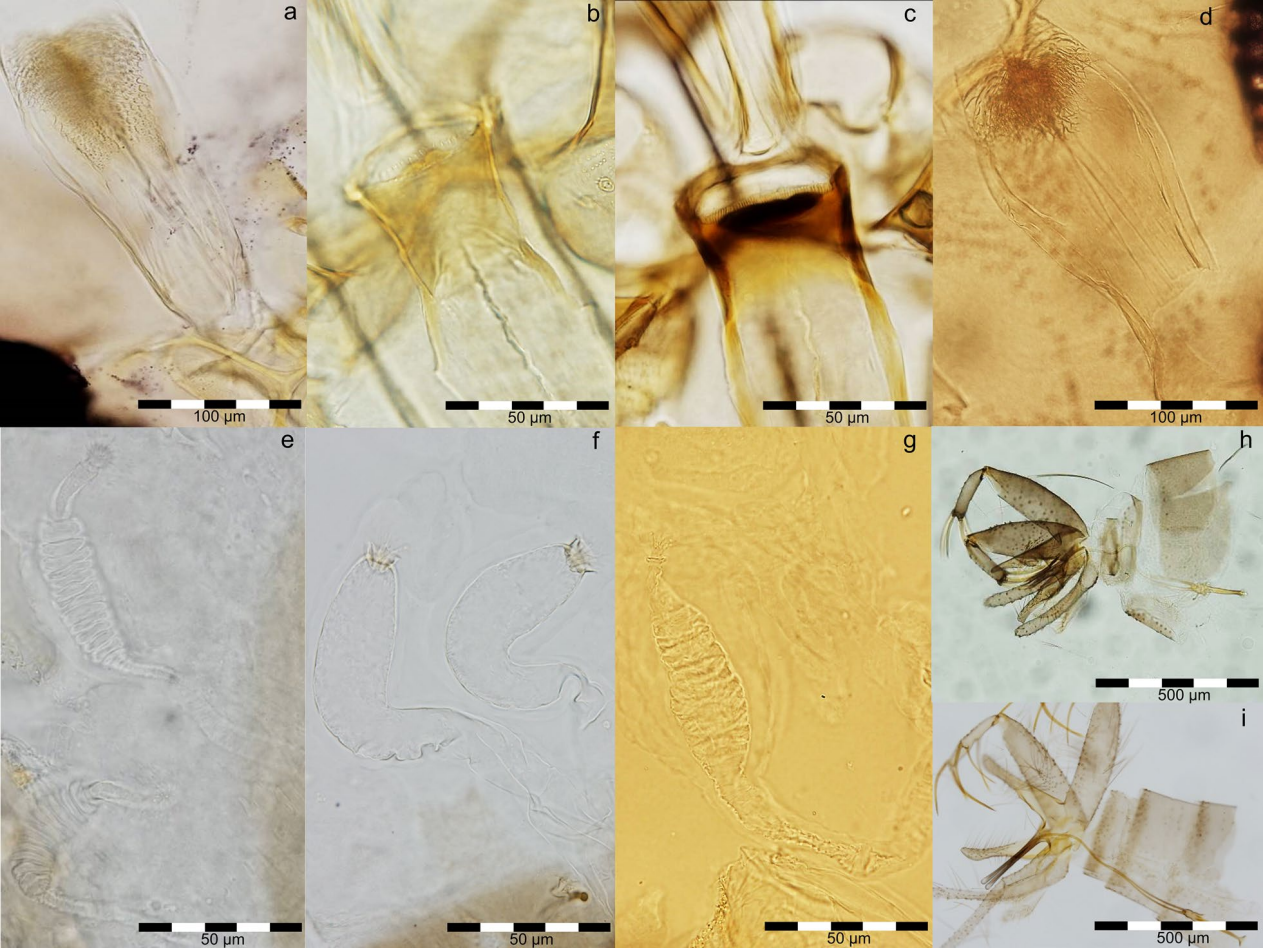
Morphological details of the three sand fly species. **a** Pharynx of *Phlebotomus neglectus*. **b** Cibarium of male *Sergentomyia minuta*. **c** Cibarium of female *Se. minuta*. **d** Pharynx of *Ph. balcanicus*. **e** Spermathecae of *Ph. neglectus*. **f** Spermathecae of *Se. minuta*. **g** Spermathecae of *Ph. balcanicus*. **h** Aedeagus *Se. minuta*. **i** Aedeagus *Ph. neglectus*

**Fig. 3:**
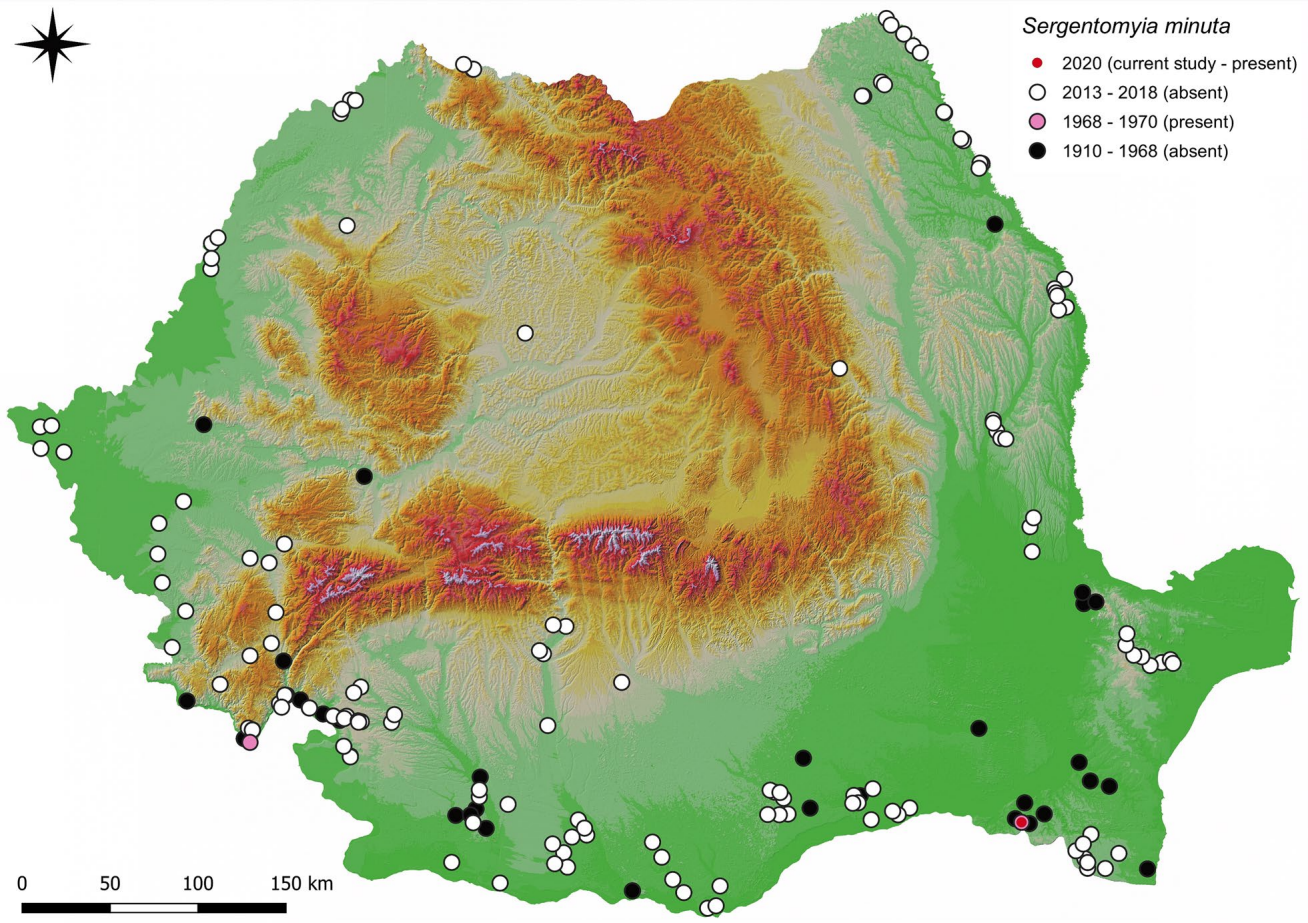
The currently known distribution areas of *Sergentomyia minuta* in Romania [3–7]. The map was generated using QGis 3.6.2 software (http://www.qgis.org)

From all (*n * =  822) collected sand flies, 99.7% (818/822) were *Ph. neglectus*, 0.1% (1/822) *Ph. balcanicus* and 0.2% (2/822) *Se. minuta* (Additional file 1: Table S1), identified by morphological characters (Fig. 2). As *Se. minuta* was trapped after a period of 50 years without records, an updated map of distribution was provided (Fig. 3). From the total number of collected individuals, the number of females (57.4%; 472/822) was statistically higher than the number of males (42.6%; 350/822) (chi-square goodness of fit, *χ*^2^  =  18.11, *P*  <  0.001) (Additional file 1: Table S1). From the total number of females, 3.6% (17/472) were engorged. No gravid females were observed.

**Fig. 4:**
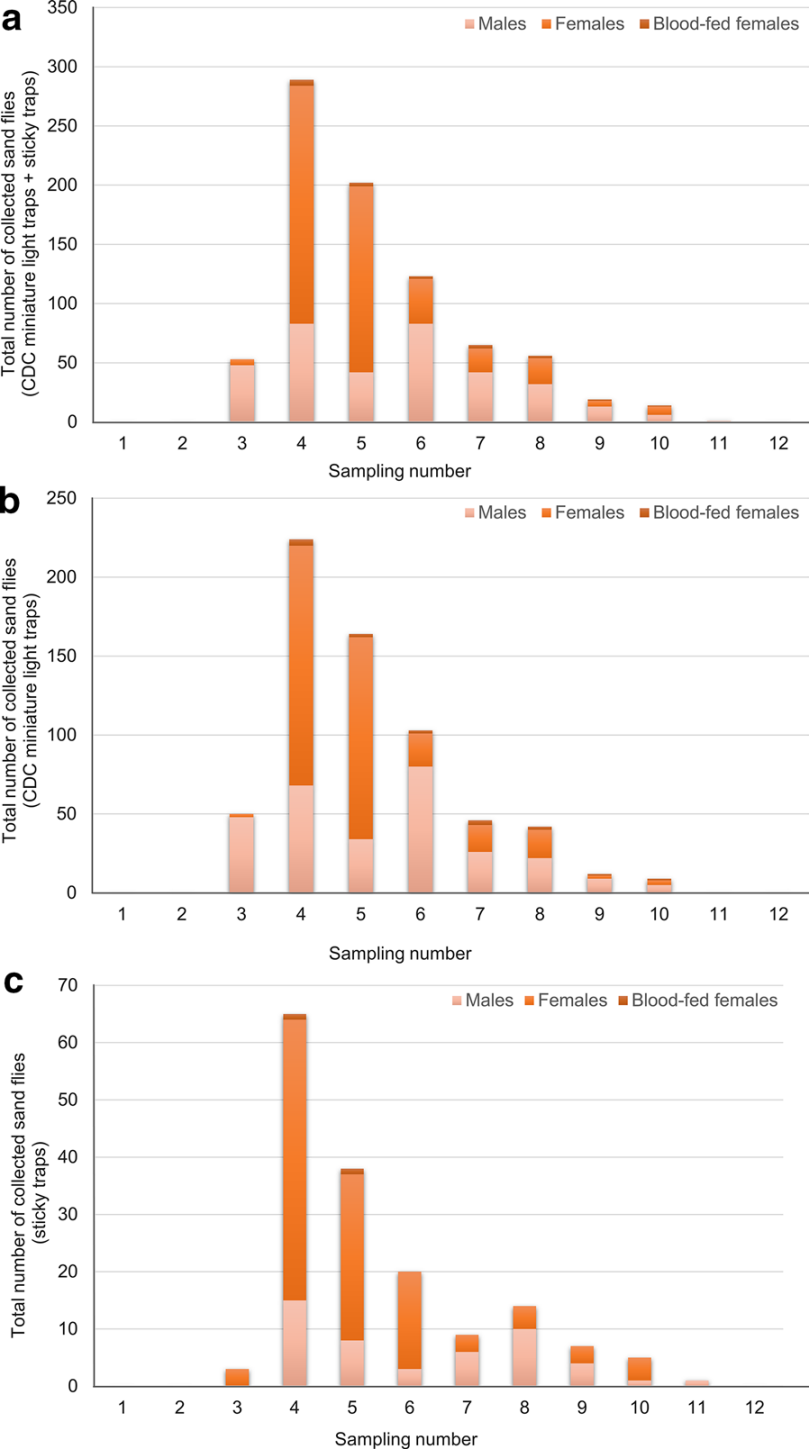
Seasonal abundance of sand flies in Canaraua Fetii (current study) (number of collected sand flies/trapping nights). **a** All traps. **b** CDC miniature light traps. **c** Sticky traps

Nine trapping periods out of 12 were positive for sand fly collection between 2 July and 24 September (Additional file 1: Table S1). The highest abundance was recorded in mid-July (16–17) (4th trapping period), accounting for 35.2% (289/822) of all captured sand flies (Fig. 4) resulting in a single activity peak (monomodal abundance trend). The total number of collected sand flies was statistically higher in the fourth, fifth and sixth trapping periods compared to the other trapping periods (Mann-Whitney *U* test, *P*  <  0.001) (Additional file 1: Table S1). Significantly more males were collected in the third trapping period than in the fourth one (Pearson’s chi-squared, *χ*^2^  =  72.49, *P*  <  0.001) and the other trapping periods (Pearson’s chi-squared*,* *χ*^2^  =  53.36, *P * <  0.001). Blood-fed females were caught in trapping periods 4–10 (Additional file 1: Table S1), with no statistical difference between trapping periods.

**Fig. 5:**
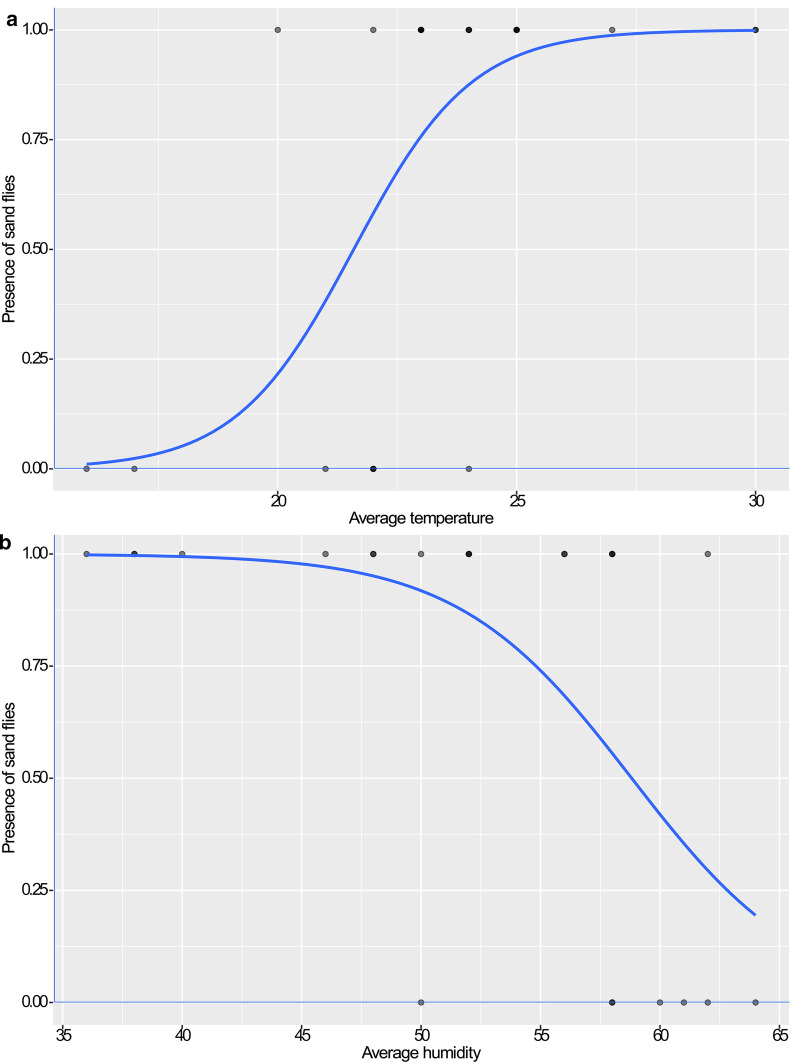
Logistic model of associations. **a** Between the presence/absence of sand flies and the corresponding average temperature values. **b** Between the presence/absence of sand flies and the corresponding average humidity values

The statistical analysis of the climatic data showed a moderate correlation between the number of captured sand flies and average temperature (Spearman’s correlation, *rs*  =  0.56, *P*  =  0.004). A strong negative correlation was also identified between the number of collected phlebotomine sand flies and the average relative humidity values of the collection day (Spearman’s correlation, *rs*  =  − 0.63, *P*  <  0.001) as well as for those of the last 5 days prior the collection day (Spearman’s correlation, *rs*  =  − 0.58, *P * =  0.003). In the two created logistic models, associations between the presence/absence of sand flies in each trapping period, the corresponding average temperature (Fig. 5a; *P* =  0.048) and average humidity values (Fig. 5b; *P* =  0.038) were identified.

Regarding the trapping type, the total number of collected specimens was statistically higher when CDC miniature light traps were used compared to sticky traps (chi-square goodness of fit, *χ*^2^  =  303.29, *P*  <  0.001). Among the total number of sand flies collected by sticky traps, the number of females (70.4%, *n*  =  114) was statistically higher than the number of males (29.6%, *n*  =  48; chi-square goodness of fit, *χ*^2^  =  26.89, *P*  <  0.001). Moreover, a significantly higher number of males were collected by CDC miniature light traps than by sticky traps (Mann-Whitney *U* test, *P * =  0.01).

## Discussion

This study presents the first detailed analysis of the seasonal activity of *Ph. neglectus* in Romania, apparently the most abundant species in the country [3]. This is the highest number of trapped individuals of *Ph. neglectus* to date in Romania [3, 4, 6, 7]. The species belongs to the *Ph. major* complex, which currently comprises five other species, widely distributed in the Old World. *Phlebotomus neglectus* is the only species of the complex present in the southern and southeastern regions of Europe [14]. In Romania, the species was historically reported in the southwestern, southern and southeastern regions of the country, along the Danube Valley and Bărăgan Plain [7, 15]. In other recent field studies, its presence was limited to the southwestern part of Romania, in Mehedinţi Plateau, where it was present mostly outdoors [3].

The present study was conducted in the nature reserve of Canaraua Fetii, previously investigated for sand fly presence, between 1968 and 1970 [7]. At that time, four sand fly species were present: *Ph. balcanicus* and *Se. minuta* (the two most abundant species) as well as *Ph. neglectus* and *Ph. sergenti*. In the present study, the diversity and abundance of the sand fly species appear to have changed in this location, with *Ph. neglectus* the most abundant species (99.7%) and a very low abundance of *Ph. balcanicus* (0.1%) and *Se. minuta* (0.2%). *Phlebotomus sergenti* was not present at the trapping period of the current study.

**Fig. 6:**
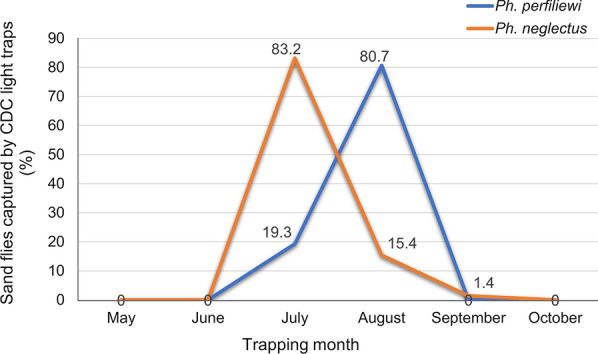
Relative abundance of *Ph. neglectus* (current study) and *Ph. perfiliewi* [4] in CDC miniature light traps

The peak activity of *Ph. neglectus* (mid-July) was approximately 15 days earlier compared to another evaluated species in Romania, *Ph. perfiliewi* (monomodal abundance trend with the peak activity at the beginning of August) (Fig. 6) [4]. The earlier and longer activity frame of *Ph. neglectus* (southeastern Romania) compared with that of *Ph. perfiliewi* (northeastern Romania) might be due to geographical position, microhabitat and climatic differences (statistically supported in both studies) [4]. In another recent study of *Ph. mascittii*, the predominant sand fly species in Central Europe, a monomodal abundance trend was also present in Austria, with variations of the peak activity in July and August [16]. Located at a similar latitude as Romania, the sand fly activity in Austria started earlier, by late June, and ended earlier, by the end of August [16]. A monomodal abundance trend was also observed for other sand fly species, such as *Ph. ariasi* in France and *Ph. kandelaki* and *Ph. balcanicus* in Georgia [17].

Considering the Balkan region, a recent review revealed that *Ph. neglectus* was also the most abundant species in 2014 and 2016 in Serbia, Kosovo, Bosnia and Herzegovina, Montenegro, Croatia and Slovenia and the second most abundant species in Bulgaria and North Macedonia [18]. The abundance trend for *Ph. neglectus* can also be bimodal (two peaks of activity), a main characteristic when the species is located in the warmer, Mediterranean climate (e.g. Greece or Cyprus) [17]. For other sand fly species, up to three density peaks and substantially longer activity periods were observed in countries with lower latitudes, such as Portugal or Turkey [17].

*Sergentomyia minuta* was the second most abundant sand fly species present between 1968 and 1970 in Canaraua Fetii [7]. In the present study conducted 50 years later in the same location, only two specimens of *Se. minuta* (one male and one female) were trapped. Between 2013 and 2018, the species was not trapped in other sampling sites in Romania, despite the trapping effort [3, 4]. A possible explanation for the apparent absence in the past years could be the trap type and the sampling date and effort. In the historical data [7], only sticky traps were used to collect sand flies, while in recent studies conducted in Romania between 2013 and 2018, only CDC miniature light traps were used [3, 4]. The two *Se. minuta* specimens (Additional file 1: Table S1) were trapped with the sticky traps, while none were trapped with the CDC miniature light traps. Other studies previously conducted in Mediterranean countries confirmed the low light attractiveness of members of the *Sergentomyia* genus [17]. Regarding the sampling date, it appears that *Se. minuta* was active and present at the beginning of July, while most of the trapping effort conducted in Romania so far was focused on the end of July to beginning of August [3]. The chosen trapping period was based on several available data: another seasonality study of *Ph. perfiliewi* conducted in Romania was assessed, and the results showed the peak activity at the beginning of August [4]. The same species, *Ph. perfiliewi*, was active only after the average minimum temperature for the previous 7 days was > 15 °C [4]. In another study, under laboratory conditions, another species, *Ph. papatasi* (also recorded in Romania), showed no larval and pupal development at 15 °C and a mean temperature of at least 18 °C was necessary for successful rearing [19]. In a more recent study, the earliest activity of *Ph. mascittii* was noticed after 5 consecutive days of mean temperature values > 15 °C and minimum temperature values > 10 °C [16].

In another study from eight Balkan countries (some of them neighboring Romania), *Se. minuta* appeared to be one of the less abundant sand fly species in Bulgaria, North Macedonia, Serbia, Kosovo, Bosnia and Herzegovina, and Montenegro. In Slovenia, the species was not present in the evaluated trapping sites. On the other hand, in Croatia the species appeared to be the second most abundant species [18].

The third sand fly species present in the current study, *Ph. balcanicus*, was one of the two most abundant species in Canaraua Fetii between 1968 and 1970 [7]. *Phlebotomus balcanicus* was also identified in North Macedonia, Serbia, Kosovo, Montenegro [18], Turkey, Armenia and Georgia [17].

The changes in the sand fly composition in Canaraua Fetii might have been influenced by a series of factors, such as: (i) demographic and human behavioral changes; (ii) changes in the host species availability; (iii) ecological changes; (iv) climatic changes; (v) use of insecticides. The selected study area (Canaraua Fetii Natural Reserve) has had a tortuous path to becoming a protected area [20]. In the past, it was a working stone quarry. Human activity was present, providing domestic hosts. The domestic animal host availability has transitioned to wildlife hosts since the stone quarry closed. This might have influenced the changes in sand fly fauna. Although the site is a protected area, a monastery was opened in 2012, and tourist pilgrimages have started. Being a protected natural area, the abundance of animal species attracts a significant number of outdoor enthusiasts. As for the insecticide use, DDT was used in Romania between 1958 and 1964 [6]. There are no data indicating aerial spraying over this location, but since the previous sand fly sampling (1968–1970) in the area was done after the DDT campaign, we consider that this is less likely to be the cause of sand fly species composition change.

## Conclusions

To our knowledge, this is the first study conducted in Romania to evaluate the seasonal activity of *Ph. neglectus*, one of the vector species of *L. infantum*, at 6-month intervals. In the reinvestigated natural habitat, the diversity of the sand fly species appears to have changed, with the predominance of *Ph. neglectus* instead of *Ph. balcanicus* and *Se. minuta*. The sand fly activity in this particular cave microhabitat appears to be longer than in other regions in Romania (July–September). A longer sand fly activity period increases the disease transmission risk to both humans and animals. The peak density of *Ph. neglectus* was observed in the middle of July, providing scientific data that can be further used during awareness campaigns for veterinarians and public health professionals. More seasonality studies are necessary to establish whether the present activity pattern of *Ph. neglectus* might have variations in other geographical areas, as the present data could not be generalized to the entire Romanian territory.

## Supplementary Information

**Additional file 1: Table S1. **Sampling data and sand fly species results of the current study.

**Additional file 2: Table S2. **Records of the climatic parameters for each day of the sampling period included in the current study.

## Data Availability

All data generated or analyzed during this study are included in this published article and its Additional files.

## Abbreviations

VL: Visceral leishmaniasis
CanL: Canine leishmaniasis
RNMA: Romanian National Meteorological Administration
